# Population and allelic variation of A-to-I RNA editing in human transcriptomes

**DOI:** 10.1186/s13059-017-1270-7

**Published:** 2017-07-28

**Authors:** Eddie Park, Jiguang Guo, Shihao Shen, Levon Demirdjian, Ying Nian Wu, Lan Lin, Yi Xing

**Affiliations:** 1 Department of Microbiology, Immunology & Molecular Genetics, University of California, Los Angeles, Los Angeles, CA 90095 USA; 2 Department of Microbiology & Parasitology, Medical School of Hebei University, Baoding, Hebei Province 071002 China; 3 Department of Statistics, University of California, Los Angeles, Los Angeles, CA 90095 USA

**Keywords:** RNA editing, Transcriptome, RNA-seq, Genetic variation, GWAS

## Abstract

**Background:**

A-to-I RNA editing is an important step in RNA processing in which specific adenosines in some RNA molecules are post-transcriptionally modified to inosines. RNA editing has emerged as a widespread mechanism for generating transcriptome diversity. However, there remain significant knowledge gaps about the variation and function of RNA editing.

**Results:**

In order to determine the influence of genetic variation on A-to-I RNA editing, we integrate genomic and transcriptomic data from 445 human lymphoblastoid cell lines by combining an RNA editing QTL (edQTL) analysis with an allele-specific RNA editing (ASED) analysis. We identify 1054 RNA editing events associated with *cis* genetic polymorphisms. Additionally, we find that a subset of these polymorphisms is linked to genome-wide association study signals of complex traits or diseases. Finally, compared to random *cis* polymorphisms, polymorphisms associated with RNA editing variation are located closer spatially to their respective editing sites and have a more pronounced impact on RNA secondary structure.

**Conclusions:**

Our study reveals widespread *cis* variation in RNA editing among genetically distinct individuals and sheds light on possible phenotypic consequences of such variation on complex traits and diseases.

**Electronic supplementary material:**

The online version of this article (doi:10.1186/s13059-017-1270-7) contains supplementary material, which is available to authorized users.

## Background

RNA editing is a prevalent post-transcriptional regulatory process that adds an additional layer of complexity to the transcriptome. In mammals, the most common form of RNA editing is A-to-I RNA editing, in which adenosine is deaminated to inosine by the *ADAR* family of enzymes [1]. Mice lacking *Adar* (also known as *ADAR1*) die embryonically at approximately embryonic day 12.5 [2, 3] while mice lacking *Adarb1* (also known as *ADAR2*) die shortly after birth due to seizures [4]. Double-stranded RNA (dsRNA) is a required substrate for *ADAR* enzymes [5] and one hypothesis states that the ancestral function of *ADARs* may have been to combat viral dsRNAs [6]; however, many groups have reported a pro-viral effect of *ADARs* [7], which may indicate a commandeering of cellular machinery that was originally anti-viral. Recent studies using mouse models show that *ADAR1* plays a central role in mammalian innate immunity by down-regulating immune response to endogenous dsRNA [8, 9]. There have been numerous reports of functional consequences of RNA editing. Earlier reported consequences involve nonsynonymous protein coding substitutions [10] and alternative splicing [11]. However, human RNA editing sites have been found to be most prevalent in Alu repeats located in non-coding regions, such as in introns and UTRs [12], which suggests possible regulatory roles of RNA editing. Indeed, nuclear retention [13], miRNA biogenesis [14], and miRNA targeting via editing of miRNA seed regions [15] or target sequences in mRNA [16] are some of the functional consequences that have been described for RNA editing in non-coding regions. Additionally, RNA editing has been shown to be associated with many diseases such as cancer [17], viral infection [18], and neurological disorders [19]. A-to-I changes in RNA lead to A-to-G changes in sequencing data because inosine is interpreted as guanosine by the reverse transcriptase. With the advent of high-throughput RNA sequencing (RNA-seq), the catalog of identified RNA editing sites has expanded tremendously [20–22], with some estimates being over a hundred million sites within most genes of the human genome [12]. Although many RNA editing sites have been identified, much less is known about how RNA editing is regulated, as well as the extent of *cis* variation and phenotypic association of RNA editing in human populations.

Quantitative trait loci (QTL) analysis has been successfully used to identify *cis*-regulatory mechanisms of quantifiable phenotypes such as gene expression (eQTL) [23] and alternative splicing (sQTL) [24]. These loci have been used to bridge the gap in our understanding between complex diseases and their respective susceptibility loci. Mapping QTLs involves testing for correlations between genomic polymorphisms and quantitative phenotypes. In addition to eQTL and sQTL analysis, other molecular traits have been studied with a QTL approach such as DNA methylation (meQTL) [25], chromatin accessibility (dsQTL) [26], and transcription factor binding (bQTL) [27]. For molecular traits corresponding to genomic loci, *cis*-QTLs are defined as significant polymorphisms that are located sufficiently close to the loci while *trans*-QTLs are defined as polymorphisms located beyond a fixed (often arbitrary) distance or on a separate chromosome. Similar to QTL analysis, allele-specific analysis has been used to investigate *cis*-regulation of gene expression [28] and RNA processing [29].

To the best of our knowledge, RNA editing quantitative trait loci (edQTL) analysis has only been applied to mouse [30] and fly [31], while allele-specific RNA editing (ASED) analysis has not been explored in any organism. In order to investigate *cis* variation of RNA editing in human populations, here we apply a comprehensive edQTL and ASED analysis to 445 lymphoblastoid cell lines (LCLs) from multiple ethnic groups and identified 1054 RNA editing sites that show significant evidence of population and allelic variation. We find that many of these edQTL and ASED signals are associated with genome-wide association study (GWAS) signals of complex traits and diseases. Lastly, we provide evidence that many *cis* SNPs associated with changes in RNA editing may regulate editing via effects on RNA secondary structure.

## Results

### RNA editing variability across 445 human LCLs

**Fig. 1:**
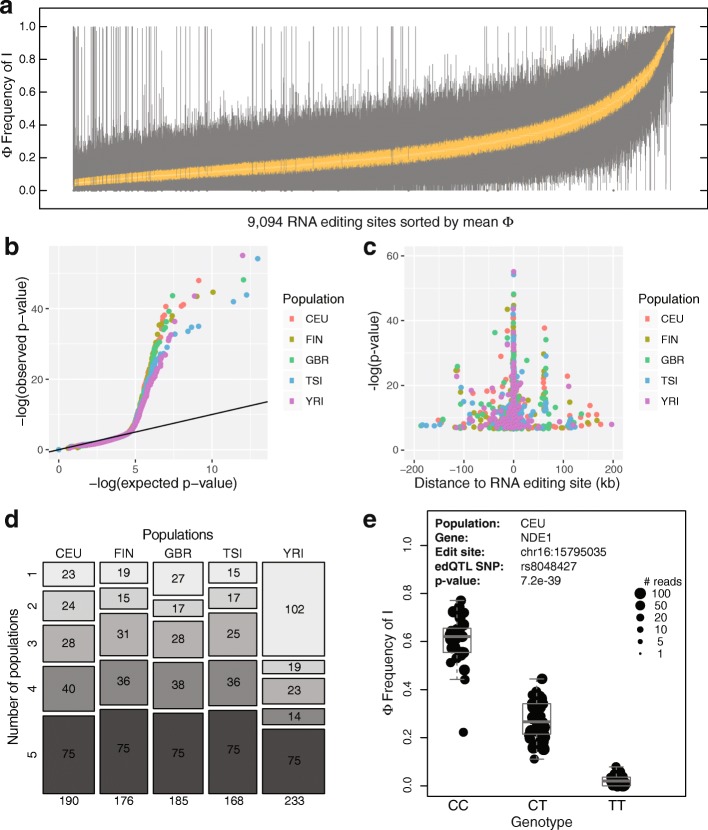
edQTL analysis to identify *cis*-regulated RNA editing events. **a** Distribution of RNA editing levels (Φ) across the 445 human LCLs. Box plots of RNA editing levels for 9094 candidate sites across 445 individuals. Sites are sorted by the mean Φ value on the *x-axis*. The inner quartile ranges for each box plot are represented in *yellow* and the medians are in *white*. **b** Quantile-quantile plot (qq-plot) testing association of RNA editing levels with *cis* genetic polymorphisms in five populations. **c** Relationship between edQTL significance and distance of SNP to editing site in five populations. Note that the apparent spikes at +60 kb and −110 kb are due to multiple RNA editing sites in a single gene (SLC35E2 for +60 kb and HLA-G for −110 kb) with edQTL signals in multiple populations. **d** Mosaic plot indicating the number of edQTL RNA editing sites shared between five populations. Values in the *top rectangles* represent population-specific edQTL sites and values in the *bottom rectangles* represent edQTL sites shared across all five populations. **e** Example of an edQTL signal in the *NDE1* gene. Box plot showing the significant association of rs8048427 with the editing level (Φ) at chr16:15795035 within the CEU population. Each *dot* represents data from a particular individual and the size of the dot indicates the number of reads covering the RNA editing site in that individual

In order to assess the extent to which natural genetic polymorphisms within human populations affect RNA editing levels, we used RNA-seq data from the Geuvadis RNA-seq Project [32] coupled with genotype data from the 1000 Genomes Project [33]. We used matching transcriptome and genotype data from LCLs of 445 individuals across five populations (CEU, FIN, GBR, TSI, YRI; Additional file 1: Table S1) to determine the association between genetic polymorphisms and RNA editing levels. Four European (CEU-Utah, FIN-Finland, GBR-Britain, TSI-Italy) and one African (YRI-Nigeria) populations are represented in the Geuvadis dataset. We limited our analysis to annotated RNA editing sites within the RADAR RNA editing database [34]. In order to identify potential RNA editing sites regulated by *cis* polymorphisms, we applied a preliminary set of filters to the ~2.6 million annotated RADAR RNA editing sites and collected 9094 candidate sites for downstream analyses. Briefly, we required the sites to have a minimum average coverage of at least two reads supporting the edited version (i.e., “I”), a minimum average total coverage of ten reads, and a minimum of 10% difference between the editing level of the 90% quantile and the 10% quantile across all 445 individuals. Within these sites, we found that RNA editing can be variable among different individuals (Fig. 1a) and hypothesized that genetic variation may account for some of the RNA editing variation. For simplicity, we introduce the term Φ (FI, frequency of inosine) to denote the RNA editing level.

### edQTL analysis

The first approach we used to test the association between RNA editing levels and genomic SNPs was with an edQTL analysis. Specifically, we tested associations between SNPs and RNA editing levels (Φ) using a generalized linear mixed model GLiMMPS [24], which accounts for coverage variation and noise in the RNA-seq data. Rather than treating the RNA-seq estimate of Φ as a point estimate, the GLiMMPS model uses the read counts for the edited and unedited transcripts to model the estimation uncertainty of the RNA editing levels. Of note, GLiMMPS was originally developed in our previous work to test association between SNPs and alternative splicing levels [24], but as a generic statistical model for QTL analysis on isoform ratio estimated from sequence count data, it is readily applicable to edQTL analysis. In order to focus on *cis*-effects, we limited our analysis to SNPs within 200 kb of the RNA editing site. Association tests were done independently for each of the five populations (CEU, FIN, GBR, TSI, YRI). We found that a significant number of RNA editing events were quantitatively associated with genomic polymorphisms (Fig. 1b). As expected, there was a higher statistical significance and greater association with SNPs that were closer to the RNA editing site (Fig. 1c). From this analysis, we identified 393 unique RNA editing sites associated with at least one edQTL SNP across the five populations at a false discovery rate (FDR) threshold of 10% (Fig. 1d; Additional file 2: Table S2). We detected 75 significant edQTL signals in all five populations, while the YRI African population had the highest number (102) of population-specific edQTLs observed only in that population. An example of an RNA editing event that is strongly associated with a genetic polymorphism occurs at chr16:15795035 (hg19) within the *NDE1* gene in which the C-allele for rs8048427 is associated with a high level of RNA editing while the T-allele nearly abolishes RNA editing (Fig. 1e). The average editing levels for the CC, CT, and TT genotypes were 60, 28, and 2%, respectively. To rule out artifacts due to unknown SNPs at RADAR RNA editing sites, we sequenced the genomic DNA around this RNA editing site in *NDE1* as well as additional sites of three other genes across multiple individuals and found no evidence of A/G polymorphism in the genome (Additional file 3: Figure S1).

### ASED analysis

**Fig. 2:**
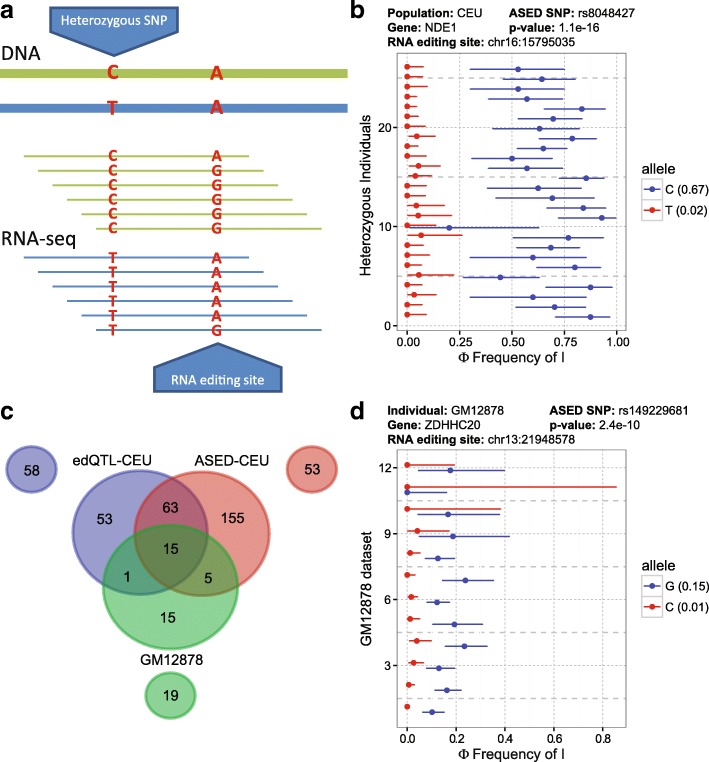
ASED analysis to identify *cis*-regulated RNA editing events. **a** Schematic diagram of ASED analysis. Heterozygous SNPs are used to assign RNA-seq reads to specific alleles. **b** Example of allele-specific RNA editing in the *NDE1* gene. ASED analysis of RNA editing site chr16:15795035 with respect to heterozygous SNP rs8048427. **c** *Cis*-regulated RNA editing sites in the CEU population. edQTL and ASED of CEU as well as multiple replicates of GM12878 were used. The three *circles* outside of the Venn diagram represent RNA editing sites that were not considered in the other two analyses due to preliminary filters and method-specific limitations. **d** Example of a *cis*-regulated RNA editing site in *ZDHHC20* associated with a rare variant, called with ASED analysis of multiple RNA-seq replicates from one individual, *GM12878. Error bars* represent likelihood-ratio test-based 95% confidence intervals of RNA editing levels inferred from read counts. Average allelic Φ values are represented in *parentheses*

To complement the above edQTL analysis, we adopted a second approach to interrogate the *cis*-variation of RNA editing through an ASED analysis (Fig. 2a). Heterozygous SNPs near RNA editing sites can be used to assign RNA-seq reads to two different alleles and Φ, the frequency of inosine, can be measured for each allele. This allows for a paired replicate statistical analysis, which aggregates ASED signals across multiple individuals sharing a given heterozygous SNP to provide greater statistical power in detecting ASED events (“Methods”). As a proof of concept analysis, we applied the ASED analysis to the RNA editing site chr16:15795035 with respect to rs8048427 (the edQTL example within the *NDE1* gene from Fig. 1e). There was a strong agreement between the edQTL result and the ASED result (Fig. 2b). The C-allele had an average Φ of 67% and the T-allele had an average Φ of 2%, which were comparable to the values for the homozygous CC and TT genotypes in the edQTL analysis (Fig. 1e).

In order to compare and contrast the edQTL and ASED approaches we performed a systematic comparison (Fig. 2c) between the CEU edQTL, the CEU ASED, as well as an ASED analysis on 12 distinct RNA-seq biological replicate samples of GM12878 [35], a member of the CEU population that was not included in the Geuvadis RNA-seq project. Each of the three approaches had different pre-processing steps and filtering criteria which meant that certain significant sites were only analyzable by one of the three approaches. In order to make a fair comparison, we excluded these sites from the comparison and represent them in the three outer circles in Fig. 2c. Sites represented in the inner Venn diagram represent sites that were included in the post-processing statistical analysis for at least two approaches and called significant by at least one approach. The CEU edQTL analysis had 132 significant sites while the population level ASED analysis in CEU had 238 significant sites; 78 significant sites were shared between the two approaches. Within these 78 shared sites, only 15 were shared with the individual level ASED analysis in GM12878, in most cases due to lack of heterozygosity in GM12878. For example, the RNA editing site chr16:15795035 in *NDE1* (Figs. **1**e and **2**b) was significant in both edQTL and population level ASED analyses but absent in the individual level ASED analysis because the GM12878 individual is homozygous for the T-allele at SNP rs8048427. We should note that although the individual level ASED analysis is limited by the availability of heterozygous SNPs in the particular individual, one benefit of this approach is that it can identify *cis*-regulated RNA editing events that are associated with rare variants. For example, the ASED SNP (rs149229681) in *ZDHHC20* is a rare variant within the CEU population with a minor allele frequency of 1%. However, the effect of *cis*-regulation of RNA editing site chr13:21948578 can be observed reproducibly with 12 RNA-seq replicates from one individual (GM12878) who is heterozygous for this SNP (Fig. 2d).

**Fig. 3:**
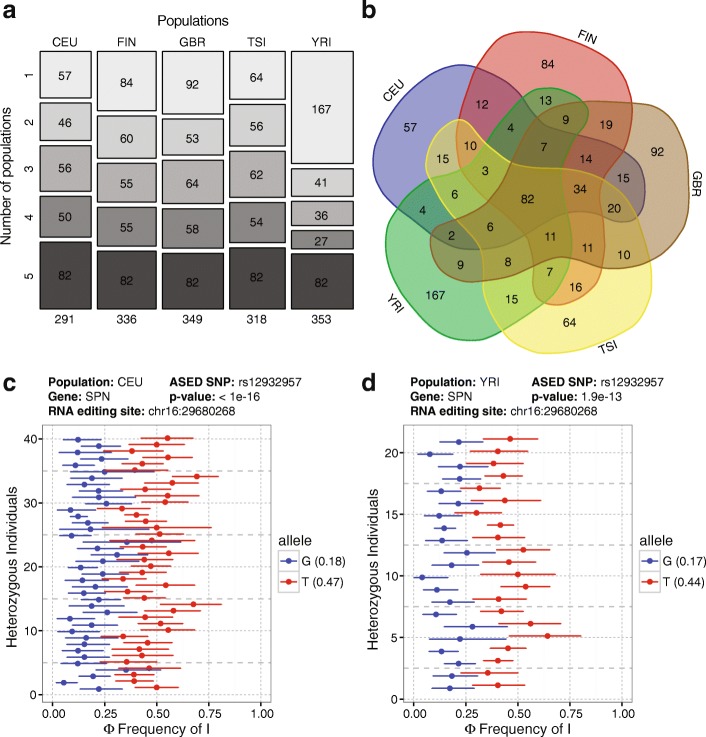
Comprehensive ASED analysis in five populations. **a** Mosaic plot indicating the number of ASED RNA editing sites shared between five populations. Values in the *top rectangles* represent population-specific ASED sites and values in the *bottom rectangles* represent ASED sites shared in all five populations. **b** The number of ASED RNA editing sites shared between five populations. Example of an ASED signal in the *SPN* gene at RNA editing site chr16:29680268 with respect to SNP rs12932957 in the CEU population (**c**) and the YRI population (**d**). *Error bars* represent likelihood-ratio test-based 95% confidence intervals of RNA editing levels inferred from read counts. Average allelic Φ values are represented in *parentheses*

We next performed population-level ASED analyses with the five populations (CEU, FIN, GBR, TSI, YRI) and obtained 826 unique ASED RNA editing sites at an FDR of 10% (Fig. 3a, b; Additional file 4: Table S3). As expected, a large proportion of ASED sites were shared between the five populations to varying degrees, with the four European populations having a higher level of shared ASED events and the YRI African population having the highest number of unique ASED events. An example of a shared ASED site (chr16:29680268) in the *SPN* gene shows the same trend of allele-specific RNA editing in the CEU population (Fig. 3c) as in the YRI population (Fig. 3d).

### Association between RNA editing and GWAS signals

**Fig. 4:**
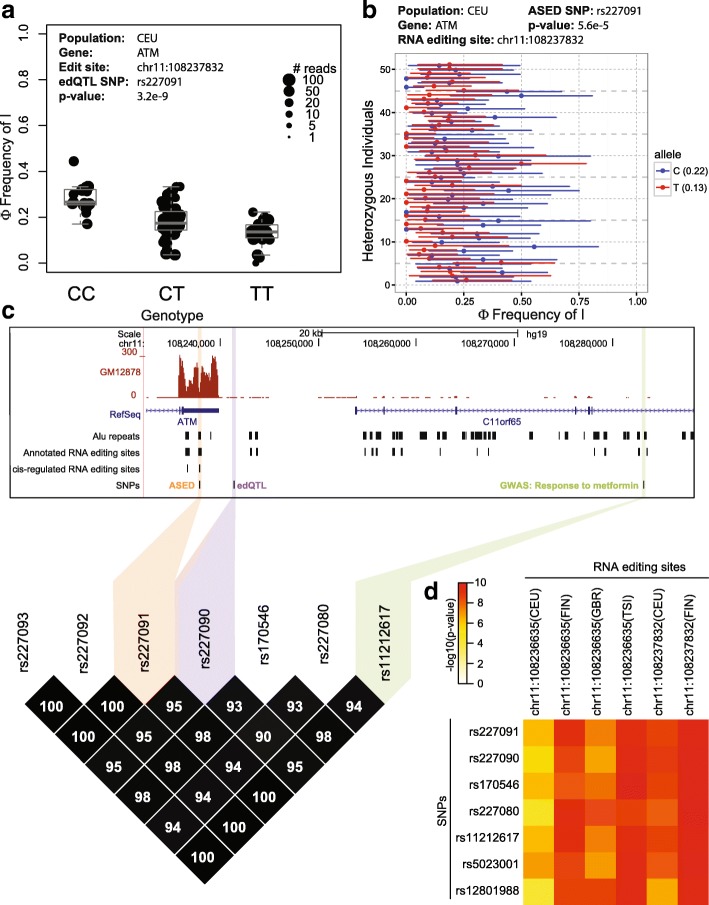
RNA editing of *ATM* is genetically associated with response to metformin. **a** Box plot showing the significant association of SNP rs227091 with editing level (Φ) at chr11:108237832 within the CEU population. Each *dot* represents data from a particular individual and the size of each *dot* indicates the number of reads covering the RNA editing site in that individual. **b** ASED allele-specific editing level (Φ) of chr11:108237832 with respect to SNP rs227091 within the CEU population. *Error bars* represent likelihood-ratio test-based 95% confidence intervals of RNA editing levels inferred from read counts. Average allelic Φ values are represented in *parentheses*. **c** LD plot showing a GWAS signal (response to metformin; *green*) linked with edQTL (*purple*) and ASED (*orange*) SNPs in *ATM*. **d** Heatmap of edQTL significance for six *cis*-regulated RNA editing sites in *ATM* along with seven *cis* SNPs. The values in the heatmap represent − log(*p* value) for the association between a given RNA editing site and a given SNP within the given population

GWAS have had much success in associating genetic variants with human traits and diseases. However, it is often unclear how the phenotype is related to the genotype. Here, we sought to assess if *cis*-regulation of RNA editing may underlie the association between certain GWAS signals and their respective traits. We tested for edQTL and ASED SNPs in high linkage disequilibrium (LD; r^2^ > 0.8 within the four European populations) with GWAS SNPs from the NHGRI GWAS Catalog [36] and identified 33 unique GWAS signals associated with *cis*-regulated RNA editing sites (Table 1; Additional file 5: Table S4). Many of these GWAS signals reflected traits and diseases known to be associated with RNA editing such as cancer, neurological traits, viral infection, and immune-related conditions. However, a surprisingly large proportion of the GWAS traits (6 of 33) were related to metabolism. One interesting example is in the 3′ UTR of *ATM* where six RNA editing sites are linked to a GWAS signal (response to metformin in type 2 diabetes) via their respective edQTL or ASED SNPs. For example, chr11:108237832, an RNA editing site with a significant signal in both the edQTL (Fig. 4a) and ASED (Fig. 4b) analyses, had a similar trend of RNA editing levels with respect to SNP rs227091, with the C-allele associated with a higher editing level and the T-allele associated with a lower editing level. *ATM* encodes for a tumor suppressor protein kinase involved in the cellular response to double-stranded DNA breaks [37]. Mutations in *ATM* occur in ataxia telangiectasia, a recessive disorder associated with radiosensitivity, cancer predisposition, immunodeficiency, and neuropathology [38]. Deficiencies in *ATM* have been linked to insulin resistance and type 2 diabetes [39]. Metformin (1,1-dimethylbiguanide) is the most commonly used drug to treat type 2 diabetes [40]. Although metformin has been clinically used since the 1950s, the exact mechanism of action has yet to be discovered [41]. The GWAS SNP rs11212617, which is associated with the effectiveness of metformin in treating type 2 diabetes [42], lies within a large haplotype block of 340 kb that encompasses genetic variants like rs227091 (Fig. 4c, d) that can affect RNA editing of the ATM gene based on our edQTL and ASED analysis.

**Table 1:** List of selected GWAS SNPs that are linked to both edQTL and ASED SNPs

Gene symbol	Editing site	ASED SNP	edQTL SNP	Linked GWAS SNP(s)	GWAS gene symbol	GWAS disease/trait	Reference (PMID)
*PSPH*	Chr7:56078339	NA	rs4947534	rs4947534	PSPH	Blood metabolite levels	24816252 [73]
	Chr7:56079087	rs4947534	NA				
	Chr7:56079100	rs4947534	NA				
*ATM*	Chr11:108236635	NA	rs12801988 rs227080 rs170546 rs5023001	rs11212617	C11orf65	Response to metformin in type 2 diabetes (glycemic)	21186350 [42]
	Chr11:108237818	rs227091	NA				
	Chr11:108237819	rs227091	NA				
	Chr11:108237832	rs227091	rs227080 rs227090				
	Chr11:108237844	rs227091	NA				
	Chr11:108237854	rs227091	NA				
*ICOSLG*	Chr21:45644472	rs8127114	rs8127114	rs4819388	ICOSLG	Celiac disease	20190752 [74]
*GINS1*	Chr20:25427805	rs6037121 rs6050623	NA	rs7267979	ABHD12	Liver enzyme levels (alkaline phosphatase)	22001757 [75]
	Chr20:25427815	rs6050626 rs6050623	rs2258728				
	Chr20:25428294	rs1047171 rs6050626 rs6037121	NA				
	Chr20:25428308	rs6050626	NA				
	Chr20:25428320	rs6050626 rs6050623	NA				
	Chr20:25428646	rs1047171	NA				
	Chr20:25428669	rs1047171	NA				
	Chr20:25428724	rs1047171	NA				
	Chr20:25428750	rs6037121	NA				
*CCL28*	Chr5:43380817	NA	rs7706402	rs11951515	CCL28	Metabolite levels (X-11787)	23934736 [76]
	Chr5:43381564	rs7706402	NA				
*FAM129A*	Chr1:184761188	rs492126 rs682331	rs570441 rs682331	rs682331	FAM129A	Obesity-related traits	23251661 [77]
	Chr1:184762487	rs526024	NA				
	Chr1:184762590	rs492126	NA

**Fig. 5:**
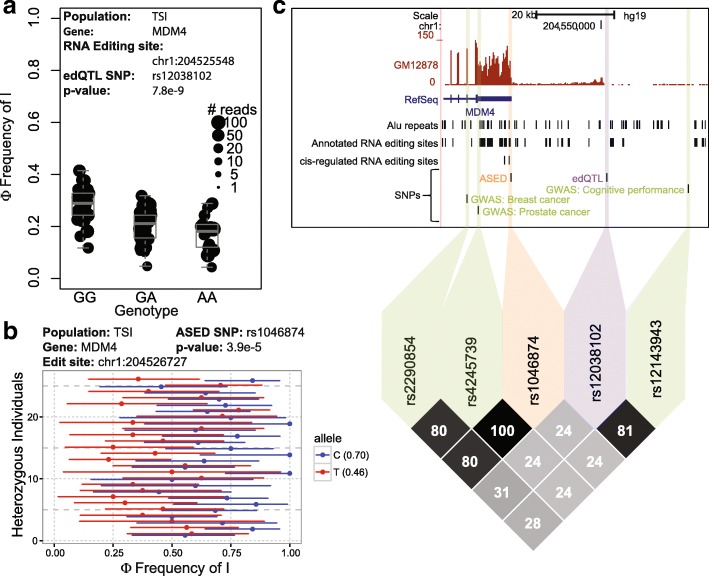
RNA editing of *MDM4* is genetically associated with cancer and cognitive performance. **a** Box plot showing the significant association of SNP rs12038102 with editing level (Φ) at chr1:204525548 within the TSI population. Each *dot* represents data from a particular individual and the size of each *dot* indicates the number of reads covering the RNA editing site in that individual. **b** ASED allele-specific editing level (Φ) of chr1:204526727 with respect to SNP rs1046874 within the TSI population. *Error bars* represent likelihood-ratio test-based 95% confidence intervals of RNA editing levels inferred from read counts. Average allelic Φ values are represented in *parentheses*. **c** LD plot showing GWAS signals (breast cancer, prostate cancer, and cognitive performance; *green*) linked with edQTL (*purple*) and ASED (*orange*) SNPs in *MDM4*

Another example of RNA editing sites linked to GWAS signals is in the *MDM4* gene. Our analysis identified multiple RNA editing sites with edQTL (Fig. 5a) or ASED (Fig. 5b) signals. One RNA editing site, chr1:204525548, was linked with SNP rs12038102 in the edQTL analysis (Fig. 5a) and this SNP was linked with another SNP rs12143943 which was reported as a GWAS signal for cognitive performance (Fig. 5c). Another RNA editing site, chr1:204526727, was linked with SNP rs1046874 in the ASED analysis, which was linked with SNPs associated with prostate cancer [43] and breast cancer [44]. Of note, *MDM4* is widely known to play a role in cancer and has been described as a regulator of p53, an important tumor suppressor [45]. Little is known about the influence of *MDM4* on cognitive performance; however, one study has shown that *MDM4* plays a pro-survival role in neurons [46].

### Impact of *cis* variants on RNA secondary structure

The number of ADAR-mediated RNA editing sites in the human transcriptome is much greater than that in many other non-primate organisms [34]. This is primarily due to the expansion of Alu repeats across the human genome. Alu elements often insert to form inverted repeats (IRAlus) in which two adjacent Alu elements are in opposite orientation. When these IRAlus are inserted into genes and transcribed as part of mRNAs, they form dsRNA hairpins which act as preferable substrates for ADAR enzymes [47].

**Fig. 6:**
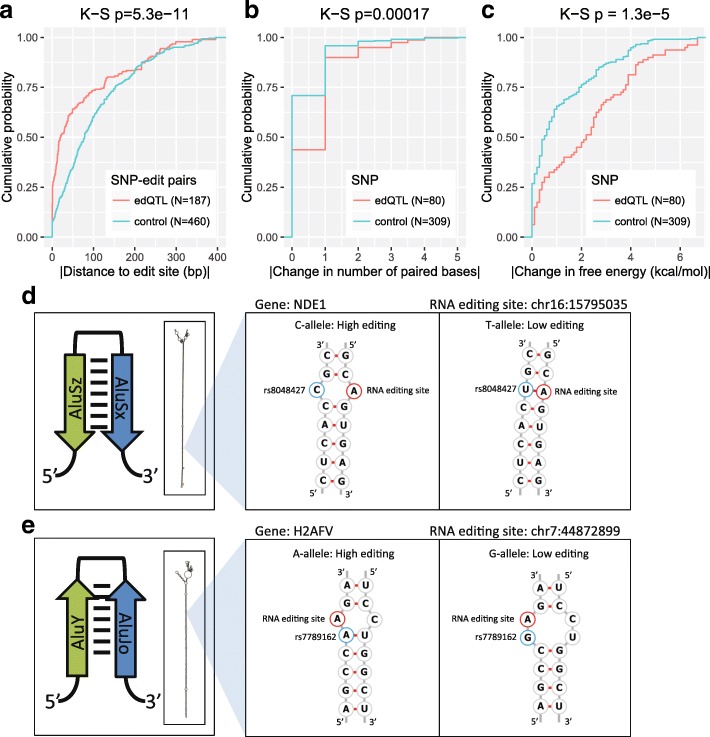
Impact of edQTL SNPs on RNA secondary structure. **a** Cumulative distribution plot comparing the absolute value of the distance between SNP–RNA editing site pairs for significant edQTL SNPs and control SNPs within the computationally predicted RNA secondary structure of the IRAlu hairpin. **b** Cumulative distribution plot comparing the absolute value of the change in the number of paired bases for significant edQTL SNPs and control SNPs. **c** Cumulative distribution plot comparing the absolute value of the change in free energy of the predicted RNA secondary structure for significant edQTL SNPs and control SNPs. The Kolmogorov–Smirnov test was used for the cumulative distribution plots. Two examples of SNPs that significantly alter RNA editing levels: SNP on the opposite Alu to the RNA editing site in *NDE1* (**d**) and SNP on the same Alu as the RNA editing site in *H2AFV* (**e**). Cartoon representation of the IRAlu hairpins and computationally predicted RNA secondary structures (*left*). Detailed base-pairing structures (*right*)

We investigated the potential effects of edQTL SNPs on RNA secondary structure of IRAlus (Additional file 6: Figure S2). Here we focused on edQTL signals because ASED signals are inherently biased towards SNPs in close proximity to the RNA editing sites within the transcripts. IRAlu sequences containing *cis*-regulated RNA editing sites were obtained and a multiple sequence alignment (MSA) was performed to identify comparable regions across multiple IRAlus (Additional file 6: Figure S2, panels 1 and 2). The alignments were sorted with respect to the RNA editing position (panels 3 and 4) and the locations of significant SNPs (*p* value <10^−10^) were plotted (panels 5 and 6). We noticed a subtle X-shape in the positional distribution of significant SNPs (panels 5 and 6), with one diagonal of the X representing SNPs located on the same Alu as the RNA editing site and the other diagonal of the X representing SNPs located on the opposite Alu to the RNA editing site. These data suggest that genetic variants spatially near the RNA editing site within the IRAlu hairpin are more likely to influence RNA editing. IRAlus can be formed from a tail-to-tail (panels 1, 3, and 5) orientation or a head-to-head (panels 2, 4, and 6) orientation, so we analyzed both types separately. Based on the predicted secondary structure of the IRAlu hairpin, we found that significant edQTL SNPs (*p* value <10^−10^) tend to be closer to the editing site than random control non-edQTL SNPs (*p* value >10^−3^) (Fig. 6a), when we considered the shortest spatial distance between the SNP and the associated editing site within the IRAlu secondary structure. Additionally, significant edQTL SNPs had a significantly larger impact on the number of paired bases (Fig. 6b) and the minimum free energy (Fig. 6c) of the predicted RNA secondary structure, suggesting that *cis* SNPs may regulate RNA editing via effects on RNA secondary structure.

We found interesting examples of *cis* SNPs that potentially influence RNA editing via RNA secondary structure (Fig. 6d, e). The *cis*-regulated RNA editing site (chr16:15795035) in the gene *NDE1* (Figs. 1e and 2b) and the associated SNP rs8048427 are located on the opposite Alu elements within an IRAlu hairpin. Here, the SNP is positioned exactly opposite to the RNA editing site within the hairpin (Fig. 6d). The C-allele is associated with a high level of RNA editing and has a C–A mismatch with the unedited site while the T-allele is associated with a low level of RNA editing and has a U–A base pairing with the unedited site. Multiple reports suggest that a C–A mismatch tends to be a favorable site for RNA editing as the mismatch would be converted into a C–I base pair by RNA editing [48]. A C–A mismatch may enhance the enzymatic reaction by facilitating a base-flipping mechanism which occurs during RNA editing [49]. When we investigated whether there was a consistent base composition preference for SNPs associated with altered RNA editing, the most striking pattern was observed at the SNP directly opposite to the RNA editing site in the IRAlu hairpin. In five out of six cases, the SNP at the opposite strand of the hairpin was a C versus T SNP and the RNA editing level was greater for the C allele than the T allele, consistent with the example in Fig. 6d for the *NDE1* gene. Another example is in the *H2AFV* gene where the SNP rs7789162 is located immediately upstream of the RNA editing site chr7:44872899 within the same Alu. The A-allele of this SNP base pairs with a U on the opposite Alu within the hairpin, leading to a single A–C mismatch across the hairpin at the RNA editing site. By contrast the G-allele creates a larger mismatch bubble involving two consecutive bases (Fig. 6e). Consequently, the A-allele is associated with high editing while the G-allele is associated with low editing, which supports the idea that the size of the mismatch bubble affects the editing level of this site.

## Discussion

We showed that the edQTL analysis and the ASED analysis are powerful yet complementary approaches to study the *cis*-variation of RNA editing. Each approach has strengths and weaknesses that complement each other, and many sites identified with one approach were not analyzable by the other approach due to certain method-specific limitations. The advantage of the edQTL approach over the ASED approach is that it is not limited to heterozygous SNPs and has no limit on distance between the SNP and the RNA editing site, as the edQTL analysis can be used to test associations with any combination of genotypes over any range. Additionally, the SNP does not need to be expressed in the transcriptome. However, the edQTL analysis may be influenced by batch effects and other non-genetic confounding factors in large-scale RNA-seq datasets [50], and cannot interrogate rare variants in the population. The main advantage of the ASED approach is that the two alleles of the same individual share the identical cellular environment. By treating the two alleles as matched pairs and multiple individuals sharing a given heterozygous SNP as replicates, a paired replicate statistical analysis can be applied to the data, which increases the statistical power and is more robust against batch effects and other confounding factors across different individuals. In fact, as we demonstrate in this work, the ASED analysis can be applied broadly across datasets generated from multiple genetically distinct individuals, or deeply across multiple replicate datasets generated from a single individual. The advantage of the latter strategy is that it can reveal *cis*-regulation of RNA editing by rare variants, as shown in the example of *ZDHHC20* (Fig. 2d). The main limitation of the ASED approach is that it relies on heterozygous SNPs that are expressed in the transcriptome and in close proximity to the RNA editing site. Additionally, incorrect phasing of heterozygous SNPs [51] or occurrence of RNA editing at an A/G SNP site in RNA can potentially result in incorrect allele assignment and confound the ASED analysis. Collectively, the integration of edQTL and ASED analyses allows us to reveal extensive population and allelic variation of A-to-I RNA editing in human transcriptomes.

One potential concern was that the RNA editing sites with significant edQTL/ASED signals were derived from unannotated genomic SNPs rather than bona fide RNA editing events. In fact, the association between SNPs and putative RNA editing sites had previously been proposed as a filter for spurious RNA editing sites in RNA-seq reads [52]. We used several strategies to assess and guard against this potential concern. First, we limited our analysis in this work to annotated SNPs and RNA editing events in HapMap and 1000 Genomes LCLs, which are the best characterized human samples with respect to genomic polymorphisms [33] and RNA editing sites [34]. Second, we sequenced the genomic DNAs of four edQTL/ASED RNA editing sites and found no evidence of A/G polymorphisms at these sites (Additional file 3: Figure S1). Lastly, if the RNA editing sites were indeed derived from genomic polymorphisms, we would expect to observe a bimodal distribution of editing level Φ concentrated at 0 and 100% in RNA-seq reads of the two alleles in the ASED analysis. Instead, we observed a skewed distribution of allele-specific RNA editing levels for significant ASED sites, in which most sites are lowly edited, which is characteristic of bona fide RNA editing sites (Additional file 7: Figure S3).

We found that many edQTL and ASED SNPs are in high association with GWAS signals, which could imply a mechanistic role of RNA editing in connecting GWAS traits with their respective genetic variants. Diverse downstream molecular processes could potentially be influenced by altered RNA editing. For instance, RNA editing has been reported to alter miRNA-mediated gene regulation [53]. Additionally, editing of IRAlus in a transcript has been suggested to alter the translation and cellular localization of the transcript [47]. Cleavage of edited transcripts is another possible downstream mechanism. For instance, *hEndoV* is a human endonuclease that is specific for inosine-containing RNAs [54].

One example of GWAS linked RNA editing events is in the *ATM* gene. *ATM* has one of the longest annotated 3′ UTRs (~3.5 kb) and it has been suggested that this allows for a rapid post-transcriptional control of gene expression in response to stimuli [55]. In addition, RNA editing in the 3′ UTR has the potential to affect miRNA-mediated regulation of tumor suppressors [16]. Thus, it is possible that RNA editing may play a role in altering the level of the *ATM* gene product and mediating a poor response to metformin for treating type 2 diabetes, although a definitive proof would require additional functional experiments. Recently, interest has also grown in the therapeutic potential for metformin to treat cancer and a number of clinical trials are in progress to determine the efficacy of metformin in cancer treatment [56, 57]. However, there have been conflicting initial reports on metformin’s effectiveness to treat cancer [58]. Since a genetic factor is associated with metformin’s efficacy in treating type 2 diabetes, it may be worth investigating whether the same variant is also associated with metformin’s efficacy in cancer treatment and whether RNA editing may mediate the therapeutic response.

We also found evidence that *cis* genetic variation could affect RNA editing levels via their effects on RNA secondary structure, extending previous reports using a smaller list of *cis*-regulated editing sites in mouse [30] and fly [31]. Specifically, we observed that SNPs associated with RNA editing levels tend to be located significantly closer to the RNA editing sites spatially within IRAlu hairpins, and may consequently alter RNA secondary structure. This change in RNA secondary structure has the potential to alter the affinity of the cellular RNA editing machinery to the substrate and subsequently change the editing level of a particular site.

In the human genome, Alu elements are the most successful retrotransposon with over a million copies, and a new Alu element is inserted in approximately one in twenty births [59]. Similar to other types of mutagenic processes during evolution, the vast majority of Alu insertions are likely non-adaptive, but some are beneficial and propagate in the population. As copies of Alu elements insert into the genome, they carry with them certain functional elements, such as transcription factor binding sites and CpG DNA methylation sites [60]. Additionally, Alu elements have many roles in the transcriptome. Transcribed Alu elements are known to interact with RNA binding proteins [61], modulate alternative polyadenylation [62] and alternative splicing [63], regulate translation efficiency [64], and contribute to the proteome [65]. Alu elements are a major contributor of endogenous dsRNAs which are targeted by the RNA editing machinery.

## Conclusions

In this work we demonstrate that RNA editing can be variable between individuals within a population and such variability can be genetically controlled. We used two orthogonal approaches (edQTL and ASED) to identify 1054 unique *cis*-regulated RNA editing sites in LCLs of 445 individuals across five populations. Given measurement limitations such as the modest RNA-seq coverage, this number is expected to be an underestimate for *cis*-regulated RNA editing events in the LCLs. Among these sites, 393 were significantly associated with edQTL SNPs and 826 were significantly associated with ASED SNPs, at an FDR of 10%. Many of these SNPs were in high LD with GWAS signals, which suggests that RNA editing may play a mechanistic role in linking genetic variation to complex traits and diseases. Additionally, we suggest a structural explanation for the causal impact of these genetic variants. Taken together, we show widespread *cis* variation of RNA editing within Alu elements and suggest that such variation may potentially contribute to phenotypic diversity across human populations.

## Methods

### Measuring RNA editing levels from RNA-seq datasets

RNA-seq alignments (hg19) for LCLs were obtained from the Geuvadis RNA-seq Project (http://www.ebi.ac.uk/Tools/geuvadis-das/) [32]. Genotype data were obtained from the 1000 Genomes Project (phase 3) [33]. Both RNA-seq and genotype data were available for 445 LCLs and these were used for subsequent analyses. A list of annotated RNA editing sites was obtained from the RADAR RNA editing database (v2) [34] and the number of RNA-seq reads supporting the edited (G in the sense of transcription) and unedited (A in the sense of transcription) sequences were obtained for each site across the 445 LCL cell lines using the mpileup command from samtools (v0.1.19) [66]. We defined the editing level, Φ (frequency of inosine), as the ratio of G reads to the sum of A and G reads $\left(RNA\; editing\; level=\frac{G}{A+G}\right)$.

### Preliminary filters of RNA editing sites for edQTL analysis

We required the RNA editing sites to meet the following criteria: a minimum average coverage of at least two reads supporting the edited version, a minimum average total coverage of at least ten reads, and a minimum of 10% difference between the editing levels of the 90% quantile and the 10% quantile across all individuals. To remove potential artifacts, we also limited our analysis to annotated RADAR RNA editing sites that did not overlap with annotated SNPs from the 1000 Genomes Project.

### edQTL analysis

For each RNA editing site, we applied the GLiMMPS statistical model [24] to SNPs within a 400-kb window centered at the editing site. The FDR was estimated using a permutation procedure [67] to obtain the null distribution of *p* values. Using five permutations, we recorded the minimum *p* value for each site over all *cis* SNPs in each permutation, and used this set of *p* values as the empirical null distribution. For a given FDR value *f*, we defined the *p* value cutoff z such that *P*(*p*_0_ < *z*)/*P*(*p*_1_ < *z*) = *f*, where *P*(*p*_0_ < *z*) is the fraction of expected *p* values from the null distribution less than *z* and *P*(*p*_1_ < *z*) is the fraction of observed *p* values from the real data less than *z*. For each editing site, the edQTL SNP was defined as the closest SNP with the most significant association. Here we used an FDR threshold of 10%.

### ASED analysis

Allele-specific alignments were obtained by aligning RNA-seq reads using STAR v2.4.2a [68] to the hg19 genome with all heterozygous SNPs N-masked, supplied with Ensembl gene annotations (release 75) using the following alignment parameters: --alignEndsType EndToEnd --outSAMattributes NH HI NM MD --outSAMtype BAM Unsorted --outSJfilterOverhangMin 8 8 8 8 8 --outFilterType BySJout --outFilterMultimapNmax 20 --outFilterMultimapScoreRange 0 --outFilterMismatchNmax 6 --outFilterIntronMotifs RemoveNoncanonicalUnannotated --alignIntronMax 300000. In-house python scripts (Additional file 8) were used to split alignments overlapping heterozygous SNPs to the two alleles. Allele-specific read counts and Φ values were calculated from the split alignments. For each replicate, we required both alleles to have non-zero coverage of RNA-seq reads and a minimum editing level of 1%. A minimum of three replicates were required for subsequent analyses. Sources of GM12878 RNA-seq data are listed in Additional file 9: Table S5.

We used a paired replicate statistical framework for reliable detection of allele-specific RNA editing signals in population-scale RNA-seq datasets. We treated the two alleles as matched pairs and multiple individuals sharing a given heterozygous SNP as replicates. We modeled and tested for the paired difference between the two alleles. Conceptually, a hierarchical framework was used to simultaneously account for the estimation uncertainty of RNA editing levels in each individual and model for the paired allelic difference in RNA editing levels across replicates. Let *Φ*_*i*1*k*_ and *Φ*_*i*2*k*_ be the editing levels of site i for allele 1 versus allele 2 in the *k*th individual. For each RNA editing site in each individual, the editing level Φ of allele 1 or allele 2 can be modeled by the counts of RNA-seq reads corresponding to the edited (*I*) and unedited (*A*) sequences via the binomial distributions:

$$ {I}_{i1k}\sim Binomial\left({n}_{i1k}={A}_{i1k}+{I}_{i1k},{p}_{i1k}={\varPhi}_{i1k}\right) $$

$$ {I}_{i2k}\sim Binomial\left({n}_{i2k}={A}_{i2k}+{I}_{i2k},{p}_{i2k}={\varPhi}_{i2k}\right) $$

We used an additive model to account for the allelic difference in RNA editing across multiple individuals. The logit transformed editing levels *logit*(*Φ*_*i*1*k*_) and *logit*(*Φ*_*i*2*k*_) can be modeled by the normal distributions:

$$ logit\left({\varPhi}_{i1k}\right)=N\left(\mu ={\alpha}_{ik},\ {\sigma}_{i1}^2\right), $$

$$ logit\left({\varPhi}_{i2k}\right)=N\left(\mu ={\alpha}_{ik}+{\delta}_i,\ {\sigma}_{i2}^2\right), $$

where the baseline editing levels common to the two alleles were represented by the fixed effect term *α*_*ik*_; the parameter *δ*_*i*_ captures the difference between the logit transformed editing levels between the two alleles; and *σ*_*i*1_^2^ and *σ*_*i*2_^2^ are the variances of allele 1 or allele 2 across multiple individuals (or replicates). The Benjamini–Hochberg procedure was used to control the FDR at 10%.

### GWAS signals

We used the NHGRI GWAS Catalog [36] (accessed 2016/03/06, v1.0) and kept SNPs with *p* values less than 10^−3^. The liftover tool from the UCSC genome browser [69] was used to convert hg38 genome coordinates of the GWAS Catalog to hg19 genome coordinates. VCFtools [70] was used to calculate linkage disequilibrium (LD) correlations between edQTL/ASED SNPs and GWAS SNPs. We required edQTL/ASED SNPs to be in high LD (r^2^ > 0.8) with GWAS SNPs. Only the four European populations were used in the LD calculation.

### RNA secondary structure prediction

RNA secondary structure prediction was preformed using RNAfold from the Vienna RNA Package [71] under its default parameters with the addition of the parameter --noClosingGU, which restricts GU pairs at the end of helices. IRAlu inverted Alu repeats were obtained by first identifying RNA editing sites within Alu repeats and then searching for the closest neighboring Alu with the correct orientation. Alu repeats without a clear inverted partner were excluded from this analysis.

### Multiple sequence alignment

For the multiple sequence alignment of the Alu sequences, we used POA (Partial Order Alignment) [72]. Alu sequences on each end of the IRAlu hairpin were aligned separately to avoid misalignments across Alu sequences. A white spacer region was placed between the two Alu sequences to facilitate the visualization of alignment results.

### Sanger sequencing of genomic DNA

A panel of 86 LCLs from the HapMap3 project was purchased from the Coriell Institute for Medical Research, Camden, NJ, USA. Three cell lines were selected for each of the three genotypes of a SNP. Genomic DNA was extracted using a Quick-DNA Miniprep Plus Kit (Zymo Research, Irvine, CA, USA).

PCR primers were designed to amplify the flanking areas of the target editing sites on the corresponding genomic DNA. Primers are: NDE1_Forward, 5′- CAACCAGGTGGAATCGTCTT-3′; NDE1_Reverse, 5′- ACTCGAACGCACCTCTAGGA-3′; ATM_Forward, 5′-CCAGGACAGCTACAGCATCA-3′; ATM_Reverse, 5′-CTAAGCCCTTCCCTTCCAAC-3′; MDM4_Forward, 5′-GTGATGGGGGATAGGGAGTT-3′; MDM4_Reverse, 5′-GCATTTCATCCCTCCTTTGA-3′; H2AFV_Forward, 5′-AGGCATGAGAATGACGTGAA-3′; H2AFV_Reverse, 5′-CTTCAACCTGGGCAAAAGAG-3′. PCR amplicons were purified by agarose gel electrophoresis and gel extraction using a PureLink® Quick Gel Extraction Kit (Invitrogen, Carlsbad, CA, USA), followed by Sanger sequencing to confirm the genomic sequence of the editing sites.

## Additional files

Additional file 1: Table S1. List of available Geuvadis LCL samples with matched RNA-seq and genotype data. (XLSX 19 kb)

Additional file 2: Table S2. List of edQTL RNA editing sites with associated SNPs. (XLSX 28 kb)

Additional file 3: Figure S1. Representative Sanger sequencing chromatograms of the genomic DNAs of four RNA editing sites to confirm absence of unannotated A/G SNPs. The RNA editing site is at the center, highlighted in *blue*. (PDF 209 kb)

Additional file 4: Table S3. List of ASED RNA editing sites with associated SNPs. (XLSX 45 kb)

Additional file 5: Table S4. List of edQTL/ASED SNPs linked to GWAS SNPs in the NHGRI GWAS Catalog. (XLSX 13 kb)

Additional file 6: Figure S2. Locations of edQTL RNA editing sites and significant SNPs within IRAlu hairpins. A diagram of the architecture of an Alu repeat is shown at the top. (panels 1 and 2). Heatmap of multiple sequence alignments (MSA) of IRAlu hairpins containing *cis*-regulated edQTL RNA editing sites. *Light blue*, *tan*, *yellow*, and *red* represent A, C, G, and T, respectively. *Dark blue* represents no aligned sequence (gaps) and *white* is an artificial spacer placed between the two Alu sequences in IRAlu hairpins. The rows of the heatmap represent individual IRAlu sequences and are sorted by the relative positions of the RNA editing sites (panels 3 and 4). The location of significant *cis* SNPs within the IRAlu hairpins are indicated (panels 5 and 6). Tail-to-tail (panels 1, 3, and 5) and head-to-head (panels 2, 4, and 6) IRAlus are analyzed separately, and the rows and columns of each group (tail-to-tail or head-to-head) of panels correspond to each other. (PDF 1540 kb)

Additional file 7: Figure S3. Histograms of RNA editing levels for heterozygous sites determined from the ASED analysis. Significant RNA editing sites were obtained from the ASED analysis and the average RNA editing level (Φ) of each allele for all heterozygous individuals was calculated. This was done for each population separately and the distribution of Φ values is plotted with respect to each allele. The allele with lower RNA editing is labeled as *Allele 1* and the allele with higher RNA editing is labeled as *Allele 2*. (PDF 104 kb)

Additional file 8: In-house python scripts for the ASED analysis. List of command-line commands and python scripts to generate allele-specific alignments along with R code for paired statistical test. (GZ 12 kb)

Additional file 9: Table S5. Data sources of 12 RNA-seq replicates of GM12878. (XLSX 11 kb)

## Abbreviations

ASED: Allele-specific RNA editing
CEU: Utah
dsRNA: Double-stranded RNA
edQTL: RNA editing quantitative trait loci
eQTL: Expression quantitative trait loci
FDR: False discovery rate
FIN: Finland
GBR: Britain
GWAS: Genome-wide association study
IRAlu: Inverted-repeat Alu
LCL: Lymphoblastoid cell line
LD: Linkage disequilibrium
miRNA: MicroRNA
MSA: Multiple sequence alignment
QTL: Quantitative trait loci
RNA-seq: RNA sequencing
SNP: Single-nucleotide polymorphism
sQTL: Splicing quantitative trait loci
TSI: Italy
UTR: Untranslated region
YRI: Nigeria
