# Supplementary material for: Population and allelic variation of A-to-I RNA editing in human transcriptomes
Source: Genome Biol. 2017 Jul 28;18:143. doi: 10.1186/s13059-017-1270-7 (PMC5532815; doi:10.1186/s13059-017-1270-7)

Additional file 3: Figure S1. Representative Sanger sequencing chromatograms of the genomic DNAs of four RNA editing sites to confirm absence of unannotated A/G SNPs. The RNA editing site is at the center, highlighted in blue.

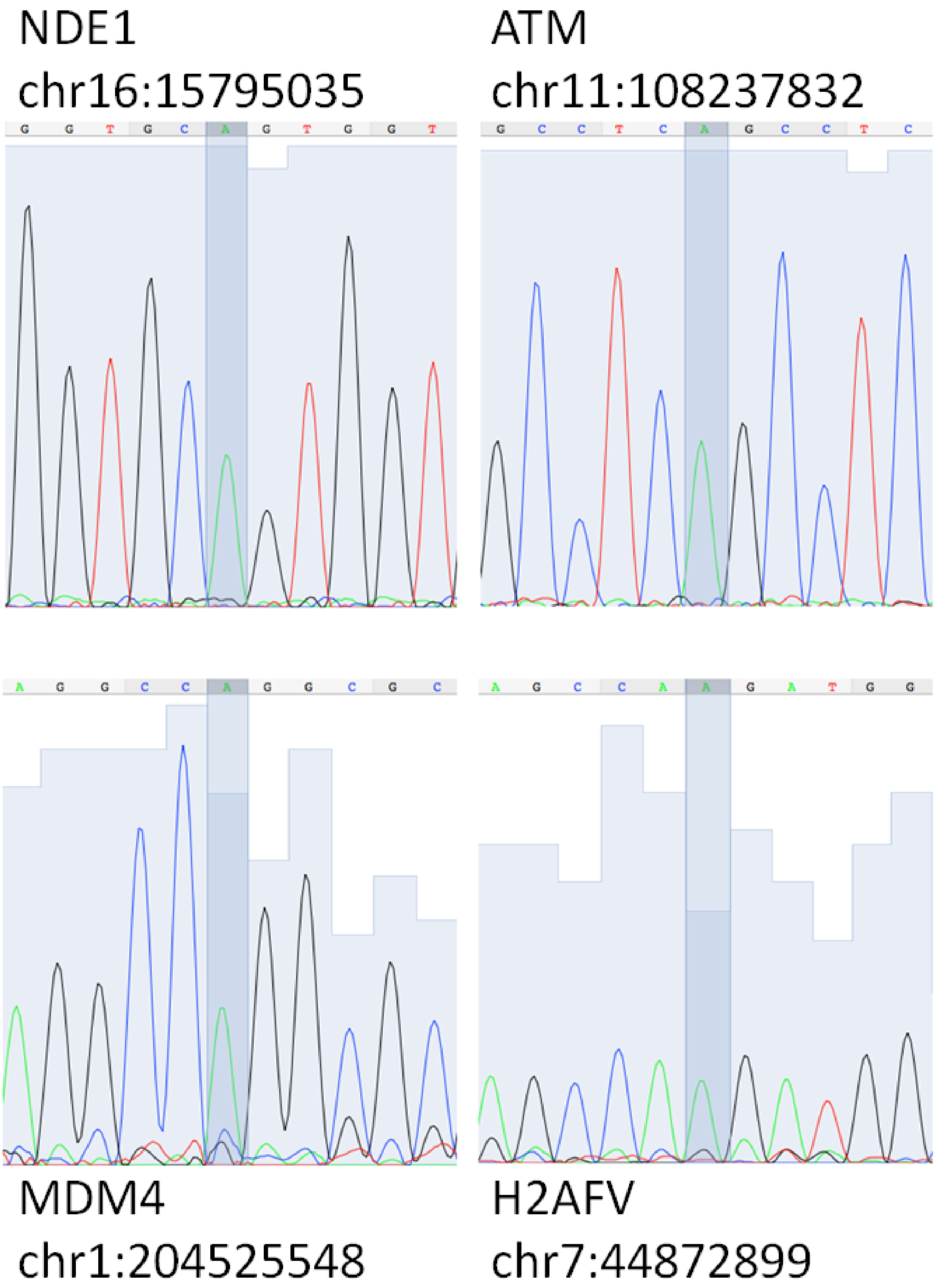

Supplement: Supplementary file 3 — Representative Sanger sequencing chromatograms of the genomic DNAs of four RNA editing sites to confirm absence of unannotated A/G SNPs. The RNA editing site is at the center, highlighted in blue. (PDF 209 kb) [file 13059_2017_1270_MOESM3_ESM.pdf]
