# Supplementary material for: Population and allelic variation of A-to-I RNA editing in human transcriptomes
Source: Genome Biol. 2017 Jul 28;18:143. doi: 10.1186/s13059-017-1270-7 (PMC5532815; doi:10.1186/s13059-017-1270-7)

Additional file 6: Figure S2. Locations of edQTL RNA editing sites and significant SNPs within IRAlu hairpins. A diagram of the architecture of an Alu repeat is shown at the top. (subpanels 1, 2) Heatmap of multiple sequence alignments (MSA) of IRAlu hairpins containing cis-regulated edQTL RNA editing sites. Light blue, tan, yellow, and red represent A, C, G, and T, respectively. Dark blue represents no aligned sequence (gaps) and white is an artificial spacer placed between the two Alu sequences in IRAlu hairpins. The rows of the heatmap represent individual IRAlu sequences and are sorted by the relative positions of the RNA editing site (subpanels 3, 4). The location of significant cis SNPs within the IRAlu hairpins are indicated (subpanels 5, 6). Tail-to-tail (subpanels 1, 3, 5) and head-to-head (subpanels 2, 4, 6) IRAlus are analyzed separately, and the rows and columns of each group (tail-to-tail or head-to-head) of subpanels correspond to each other.

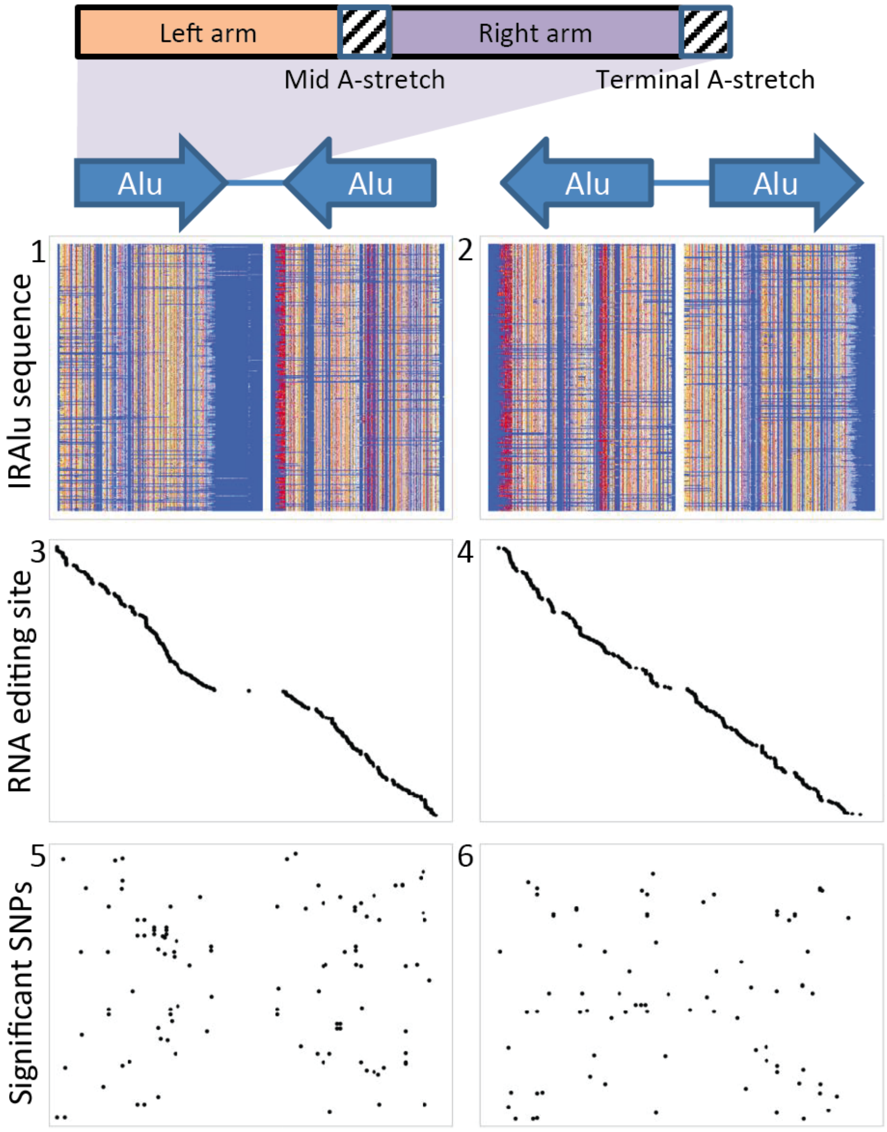

Supplement: Supplementary file 6 — Locations of edQTL RNA editing sites and significant SNPs within IRAlu hairpins. A diagram of the architecture of an Alu repeat is shown at the top. (panels 1 and 2). Heatmap of multiple sequence alignments (MSA) of IRAlu hairpins containing cis-regulated edQTL RNA editing sites. Light blue, tan, yellow, and red represent A, C, G, and T, respectively. Dark blue represents no aligned sequence (gaps) and white is an artificial spacer placed between the two Alu sequences in IRAlu hairpins. The rows of the heatmap represent individual IRAlu sequences and are sorted by the relative positions of the RNA editing sites (panels 3 and 4). The location of significant cis SNPs within the IRAlu hairpins are indicated (panels 5 and 6). Tail-to-tail (panels 1, 3, and 5) and head-to-head (panels 2, 4, and 6) IRAlus are analyzed separately, and the rows and columns of each group (tail-to-tail or head-to-head) of panels correspond to each other. (PDF 1540 kb) [file 13059_2017_1270_MOESM6_ESM.pdf]
