# Supplementary material for: Population and allelic variation of A-to-I RNA editing in human transcriptomes
Source: Genome Biol. 2017 Jul 28;18:143. doi: 10.1186/s13059-017-1270-7 (PMC5532815; doi:10.1186/s13059-017-1270-7)

**Additional file 7: Figure S3.** Histograms of RNA editing levels for heterozygous sites determined from the ASED analysis. Significant RNA editing sites were obtained from the ASED analysis and the average RNA editing level (Φ) of each allele for all heterozygous individuals was calculated. This was done for each population separately and the distribution of Φ values is plotted with respect to each allele. The allele with lower RNA editing is labeled as Allele 1 and the allele with higher RNA editing is labeled as Allele 2.

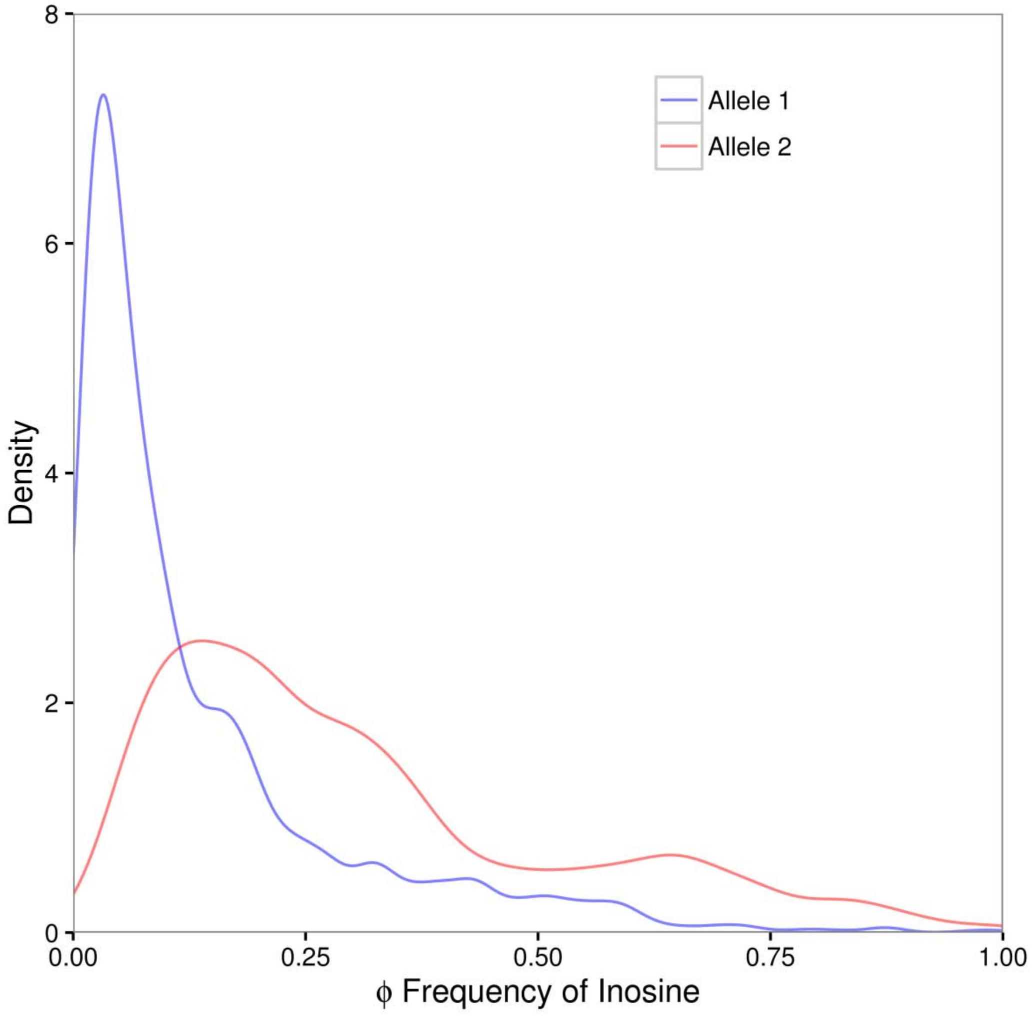

Supplement: Supplementary file 7 — Histograms of RNA editing levels for heterozygous sites determined from the ASED analysis. Significant RNA editing sites were obtained from the ASED analysis and the average RNA editing level (Φ) of each allele for all heterozygous individuals was calculated. This was done for each population separately and the distribution of Φ values is plotted with respect to each allele. The allele with lower RNA editing is labeled as Allele 1 and the allele with higher RNA editing is labeled as Allele 2. (PDF 104 kb) [file 13059_2017_1270_MOESM7_ESM.pdf]
